# The spread of mind: psychological contagion in theory and critique

**DOI:** 10.3389/fpsyg.2025.1631927

**Published:** 2025-11-21

**Authors:** James Houran, Neil Dagnall, Ram P. Sapkota, Rense Lange

**Affiliations:** 1 Integrated Knowledge Systems, Dallas, TX, United States; 2 Laboratory for Statistics and Computation, ISLA—Instituto Politécnico de Gestão e Tecnologia, Porto, Portugal; 3 Manchester Metropolitan University, Manchester, United Kingdom; 4 Department of Psychology, Faculty of Arts, University of Regina, Regina, SK, Canada

**Keywords:** behavioral mimicry, emotional contagion, suggestion and expectancy effects, memetic transmission, narrative review, psychological contagion

## Abstract

Psychological contagion (PC) involves the transfer and “snowballing” of emotions, perceptions, or behaviors within or across individuals, often through subtle, automatic, or unconscious mechanisms. This narrative review synthesizes cross-disciplinary evidence and proposes a mechanism-level Cascading-Resonance Model of PC. Drawing on neuroscience, social psychology, media studies, and diffusion theory, we identify nine mechanisms that instantiate a three-layer process involving individual resonance, interpersonal synchronization, and group-level cascade. We summarize empirical patterns across cross-modal domains, map mechanisms to observable indicators and intervention levers, as well as offer falsifiable propositions for measurement and platform-level testing. We further explore the role of contagion in modern-day controversies and anomalous experiences. Findings are preliminary and based on conceptual synthesis rather than exhaustive meta-analysis, so we highlight priority directions for causal, multilevel research and policy evaluation.

## 1 Introduction

The human psyche—including consciousness—is inherently social: shaped by and responsive to the perceptions, moods, and actions of others. Across evolutionary time, this resonance has enabled people to share attention, coordinate behavior, and navigate complex group dynamics. At the heart of this responsiveness lies the concept of *psychological contagion* (PC), i.e., the spontaneous, often unconscious, rippling or snowballing of affective, perceptual, or behavioral information within or across individuals. From mimicked smiles to mass hysteria, PC reflects how people can be influenced by others’ mental and physical states in ways that blur the line between autonomous and socially-induced experience (Wheeler, 1966; Colligan et al., 1982; Hatfield et al., 1993a,b).

This paper examines the broad PC-related literature relative to the three primary components of human experience (Eagly and Chaiken, 1993; Ajzen, 2001; Hogg and Vaughan, 2005)—(a) *emotional* (i.e., affective), (b) *perceptual* (i.e., cognitive), and (c) *behavioral* (i.e., conative)—and explores how they interrelate. Table 1 lists representative phenomena that instantiate these three forms, and in a later section we introduce cross-cutting mechanisms used to interpret them. While each domain involves some distinct mechanisms, they are unified by their capacity to inflate an individual’s experience and propagate it across social systems. To account for this proliferation, we explore how contagion potentially scales from *person-oriented effects* (e.g., priming, mimicry, suggestion) to *group-level dynamics* (e.g., mass psychogenic illness, social movements) via a proposed process in which suggestion and attention, operating through shared identity and media amplification, escalates intrapsychic shifts into collective psychological states.

**TABLE 1:** Major types of psychological contagion.

Name	Definition	References	Category
**Individual-oriented**			
Nocebo effects	Negative psychological or physiological responses to a non-harmful treatment due to expectations of adverse effects.	Benedetti, 2008	Perceptual
Placebo effects	Psychological or physiological improvement following a treatment that has no therapeutic effect, due to the belief in its efficacy.	Price, 2000	Perceptual
Conformity	The act of matching attitudes, beliefs, and behaviors to group norms, often influenced by social pressure.	Asch (1956)	Behavioral
Critical mass or adoption rates	The point at which enough individuals in a society have adopted a behavior, causing its rapid spread across the population.	Rogers, 2003	Behavioral
Memetic behavior	Behaviors or ideas that spread and evolve within a society, often analogous to the way genes replicate.	Blackmore, 1999	Behavioral
Persuasion	The process by which an individual or group influences another’s beliefs, attitudes, or behaviors through communication.	Cialdini, 2001	Behavioral
**Group-oriented**			
Collective psychoses	Widespread, typically temporary, breakdowns of cognitive and emotional functioning within a group, often manifesting as paranoia or delusions.	Danziger, 1993	Emotional
Contagion of anxiety or panic	The spread of anxiety or panic within a group or society, often triggered by external events, leading to widespread fear or irrational behavior.	Taylor, 2013	Emotional
Group or collective psychopathology	A mental disorder affecting an entire group or society, often manifesting as collective paranoia or social phobia.	Klein, 2004	Emotional
Mass or epidemic hysteria	The rapid spread of symptoms of hysteria within a group, often triggered by stressful events or societal pressures.	Kirmayer and Robbins, 1991	Emotional
Mass sociogenic illness	Illnesses or symptoms that spread rapidly through a group, typically driven by social and cultural factors rather than biological causes.	de Waal, 2016	Emotional
Moral contagion	The spread of moral beliefs, ethical standards, or political views across individuals or groups.	Haidt, 2012	Emotional
Psychic epidemics	Sudden and unexplained widespread manifestations of psychological symptoms, often without a clear medical or psychological cause.	Bartholomew and Wessely, 2002	Emotional
Social contagion	The spread of behaviors, emotions, or attitudes through social networks, often unconsciously.	Christakis and Fowler, 2007	Emotional
Social media contagion	The rapid spread of ideas, emotions, or trends through social media platforms, often with widespread visibility and influence.	Sherman and Payton, 2014	Emotional
Baader–meinhof phenomenon	The tendency for people to notice something more frequently after they first encounter it, often linked to selective attention.	Gilovich and Savitsky, 2002	Perceptual
Mass delusions	Collective false beliefs that spread among a group or society, often unsupported by evidence.	Lutz, 1988	Perceptual
Contagion of violence	The spread of violent behavior within groups, often due to imitation, media exposure, or environmental stressors.	Bandura, 2001	Behavioral
Cultural contagion	The spread of cultural norms, behaviors, or values within and between societies, influencing larger cultural shifts.	Richerson and Boyd, 2005	Behavioral
Group or mass conversion reaction	A sudden, widespread occurrence of conversion symptoms (such as blindness, paralysis) within a group, typically occurring in high-stress environments.	Kellner, 1984	Behavioral
Hysterical or behavioral contagion	The spread of hysterical symptoms or behaviors through a group, often involving imitation or emotional contagion.	Marks and Madsen, 1991	Behavioral
Mob or hooligan behavior	The rapid escalation of aggressive, destructive, or unlawful behavior within a group, often driven by deindividuation, anonymity, peer reinforcement, and emotional arousal.	Le Bon, 2002	Behavioral
Netflix and streisand effects	The phenomenon where efforts to block information or control content lead to its unintended spread or greater visibility.	Jansen and Martin, 2015	Behavioral
Viral marketing	A marketing strategy that encourages individuals to spread promotional messages or content, often through social networks.	Godin, 2000	Behavioral

Suggestion effects are well-documented across the cognitive and biomedical sciences. Experimental paradigms validate that social cues—e.g., instructions, tone of voice, or visual emphasis—can bias sequential perception, memory, or physiological responses (Wegner, 2002; Wager et al., 2004; Colloca and Miller, 2011). But PC transcends the lab: historical and contemporary cases indicate that beliefs, emotions, and marked symptoms can sweep through entire communities, echoing the structure of infectious disease or “viral” outbreaks (Colligan et al., 1982; Boss, 1997; Bartholomew and Wessely, 2002). For instance, Sapkota et al. (2014) documented a “mass possession” outbreak in rural Nepal in which direct verbal suggestion and visual exposure precipitated dissociative episodes among women. A guru’s instruction that a held lemon would “escape” if witchcraft were involved produced strong embodied reactions in highly suggestible individuals, and the phenomenon spread as witnesses who saw neighbors collapse later reported similar sensations—frequently precipitated when told it was “their turn.”

This dramatically illustrates how verbal suggestion, visual cues, and peer influence can catalyze contagion-like processes in cultural contexts, reinforcing the broader point about suggestion effects in psychological contagion (Sapkota et al., 2014). Clinical psychology often describes such events as “psychic epidemics, collective psychoses, mass delusions, or mass sociogenic illness,” whereas sociology and media studies refer to “social contagion, viral behavior, and memetic transmission” (Dawkins, 1976; Christakis and Fowler, 2013). Indeed, business, political, and health communication strategies often rely—intentionally or not—on PC principles to spread messages, encourage conformity, or shape public behavior. The concept is also implied or assumed in modern disputes over fake news, algorithmic manipulation, cancel culture, and ideological polarization. All this underscores the hot-button issues of information virality and emotional amplification in today’s “digital information age” (Kramer et al., 2014; Aral and Nicolaides, 2017).

## 2 The present approach

Despite growing cross-disciplinary interest in PC effects, the literature remains fragmented, with psychological, social, and technological mechanisms rarely integrated into a unified theoretical framework. Even though meta-analytic work (e.g., Pizarro et al., 2022) shows convergence for collective effervescence across contexts, much of the PC research still treats emotional, perceptual, and behavioral contagion in isolation rather than as interacting processes. This raises a fundamental question of whether various contagion phenomena represent manifestations of a shared, fractal-like process or reflect a collection of distinct mechanisms loosely grouped by metaphor. Accordingly, this critical narrative literature review (a) synthesizes findings across the emotional, perceptual, and behavioral contagion domains, (b) highlights cross-domain patterns and interactions, and (c) introduces a scalable “cascading-resonance” model for understanding contagion from individual to group levels. In this way, we aim to advance both theoretical clarity and practical insight into how psychological states are socially transmitted—whether through face-to-face interaction, mass media, or digital platforms.

This type of review aims to summarize and synthesize existing knowledge on a topic. It also helps to identify gaps in research, inform current practice, and guide future research (Ferrari, 2015; Greenhalgh et al., 2018; Baethge et al., 2019). Unlike systematic reviews that involve exhaustive searches and long processing times, narrative reviews are more qualitative, discursive, and flexible in structure and do not necessarily follow strict methodologies (Baethge et al., 2019). That said, some approaches may incorporate structured elements—such as simplified flow diagrams or transparent inclusion rationales—to enhance clarity and rigor (Ferrari, 2015). Also note that our review strives to go beyond mere description to include a degree of analysis and conceptual innovation, which typically produces a working hypothesis or practical model (Grant and Booth, 2009).

We therefore implemented an iterative selection, appraisal, and synthesis process to identify studies useful for cross-context model building and theory formation on perceptual, emotional, and behavioral forms of contagion. Our aim was to compile a representative, not exhaustive, literature set that combined conceptual insights and empirical findings sufficient to develop a holistic, scale sensitive model of PC. We began with technical keyword selection across relevant domains, ran systematic searches of bibliographic and gray sources, and visually inspected titles and abstracts to flag content deemed most relevant across the three contagion modalities.

We specifically mined the Google Scholar, PubMed, and Scopus databases for the most significant, representative, and topical items as judged by the authors via a visual inspection and internal discussion. Boolean logic filtered the target literature efficiently (Bramer et al., 2017) via the search strategy: “(“psychological contagion” OR “emotional contagion” OR “behavioral contagion” OR “perceptual contagion” OR “social contagion” OR “affective contagion” OR “mass hysteria” OR “moral panic” OR “shared delusion” OR “group psychosis” OR “collective behavior” OR “group influence” OR “memetic spread” OR “viral behavior” OR “media-induced panic” OR “neural mirroring” OR “mirror neurons”) AND (critique OR “critical theory” OR “cultural analysis” OR “sociocultural critique” OR “media criticism” OR “philosophical analysis” OR “epistemological critique” OR “theoretical framing” OR “reflexive analysis” OR “discourse analysis” OR “biopolitics” OR “pathologization” OR “social construction”) AND (hysteria OR rumor OR delusion OR belief OR panic OR anxiety OR emotion OR imitation OR empathy OR mimicry OR conformity OR suggestion OR influence OR transmission).” This ensured that outputs were relevant to our aims while maintaining critical depth (Boell and Cecez-Kecmanovic, 2015).

We next prioritized the candidate records by recency (published approximately within the last 20 years) and use of empirical methods (quantitative or qualitative), and we incorporated several topic areas suggested by peer reviewers that our initial search had missed. The project team then conducted iterative appraisal and discussion to finalize an inclusive set of studies for synthesis, summarized study features in a structured evidence table, and synthesized findings narratively by mapping themes, moderators, and mechanisms across experimental, observational, and qualitative streams. The final analytic move was to develop a grounded theory interpretation that integrated the mapped themes into a cohesive model describing escalating scales of PC. The full process is formally summarized below as a sequential evaluation expression:

Keyword selection → Conduct searches → Title and abstract screening for cross-modality relevance → Prioritize by recency and empirical method → Review and inclusion for representativeness → Iterative synthesis and thematic mapping → Grounded theory development and final interpretation     (1)

The remainder of this paper is organized into several sections. *First*, we introduce a concise set of cross-cutting mechanisms to serve as the analytic vocabulary for interpreting evidence across emotional, perceptual, and behavioral contagion. *Second*, we present domain-specific narrative syntheses that reference these mechanisms. Third, we summarize empirical convergence across domains and evaluate where mechanisms show consistent, partial, or weak support. *Finally*, we develop a grounded “Cascading-Resonance” model of PC that integrates these mechanisms into a multilevel account. Our review protocols were not pre-registered, but we strived to the Journal Article Reporting Standards (Kazak, 2018) and thus describe how we determined our samples, data exclusions (if any), research questions, applicable manipulations, and all measures and data abstractions used in our analyses.

## 3 Preliminaries

Table 2 presents an illustrative matrix that cross-classifies PC phenomena by *modality* (emotional, perceptual, behavioral) and *orientation* (individual-oriented vs. group-oriented), providing a compact map to use while reading the subsequent domain summaries. Our iterative synthesis and thematic mapping of the selected PC literature revealed a shared, multilevel architecture linking emotional, perceptual, and behavioral contagion: (1) *individual receptivity*—dispositional and state factors (e.g., suggestibility, heightened arousal) that lower thresholds for social influence; (2) *cue-driven alignment*—stimulus features and framings (e.g., mimicry, narrative cues, expectancy) that convert external input into internal affect or perception; (3) *rapid interpersonal feedback*—real-time social processes (e.g., social appraisal, entrainment) that amplify and coordinate responses among interactants; and (4) *structural amplification*—network, institutional, and identity-based forces (e.g., algorithmic boosting, prestige signals, moral framing) that stabilize and scale local synchrony into group-level cascades.

**TABLE 2:** Illustrative matrix of psychological contagion phenomena.

Contagion modality	Individual-oriented	Group-oriented
Emotional	Facial mimicry, empathic resonance	Group mood shifts, moral contagion, outrage mobs
Perceptual	Placebo/nocebo, frequency illusion	Mass psychogenic illness, social illusions
Behavioral	Yawning, mirroring, priming effects	Mob behavior, viral trends, suicidality clusters

These four process stages of PC ostensibly combine in varying proportions to produce multimodal chains (e.g., emotion → perception → behavior), operate across *micro* (individual), *meso* (interpersonal), and *macro* (network/institutional) levels, and provide the analytic vocabulary and testable constructs used throughout this review to explain why affect, perception, and action frequently cascade together. Our thematic mapping further identified nine cross-domain mechanisms (see Table 3 for a numbered glossary) that our review suggests drive the four process stages.

**TABLE 3:** Thematic outputs from an iterative mapping of cross-domain mechanisms of psychological contagion.

#	Contagion mechanism	Definition	References
1	Suggestibility and boundary thinness	Dispositional and state permeability that increase susceptibility across domains.	Cardeña et al., 2008; Evans et al., 2019
2	Expectancy and framing	Verbal or contextual cues that bias priors and shape perception, affect, and action.	Colloca and Miller, 2011; Friston, 2005
3	Automatic mimicry and embodied simulation	Rapid sensorimotor/facial mirroring that seeds affective and behavioral alignment.	Chartrand and Bargh, 1999; Dimberg et al., 2000
4	Attention synchronization and salience amplification	Shared focus (physical or algorithmic) that raises exposure and signal prominence.	Berger and Milkman, 2012; Kramer et al., 2014
5	Interpersonal entrainment and neural synchrony	Temporal alignment of physiological or neural activity supporting coordination.	Dumas et al., 2010; Hasson et al., 2012
6	Social appraisal and norm signaling	Interpretive and status cues that convert raw affect/perception into social meaning.	Klucharev et al., 2009; Parkinson, 2011
7	Network and institutional amplifiers	Algorithms, media, prestige, and scripts that stabilize and scale local synchrony.	Christakis and Fowler, 2013; Vosoughi et al., 2018
8	Moralization and affective intensity	Ethical framing and high arousal that increase transmissibility and resistance.	Brady et al., 2020; Haidt, 2012
9	Feedback loops and threshold dynamics	Iterative reinforcement and tipping points that produce nonlinear cascades.	Gladwell, 2000; Granovetter, 1978

Specifically, Table 3 defines Mechanism 1: Suggestibility and boundary thinness; Mechanism 2: Expectancy and framing; Mechanism 3: Automatic mimicry and embodied simulation; Mechanism 4: Attention synchronization and salience amplification; Mechanism 5: Interpersonal entrainment and neural synchrony; Mechanism 6: Social appraisal and norm signaling; Mechanism 7: Network and institutional amplifiers; Mechanism 8: Moralization and affective intensity; and Mechanism 9: Feedback loops and threshold dynamics.

We adopt the four process stages and the numbered glossary of PC mechanisms as the analytic vocabulary and set of testable constructs, with each domain subsection explicitly citing mechanism numbers (e.g., “Mechanism 4: Attention synchronization”) to ensure cohesion, enable direct cross-domain comparison, and facilitate hypothesis generation.

## 4 Exploring emotional contagion

Our review suggests that this modality mainly involves Mechanism 1: Suggestibility, Mechanism 3: Automatic mimicry, Mechanism 5: Interpersonal entrainment, and Mechanism 6: Social appraisal (see Table 3). Also known as interpersonal emotion transfer (IET), this category denotes the process of systematically or spontaneously “catching” others’ emotions (Hatfield et al., 1993a,b). Noting the importance of this phenomenon, Herrando and Constantinides (2021) recently undertook a review and explored potential future directions. Neural mirroring, facial mimicry, and empathic processes are key mechanisms, with research suggesting a link between hypnotic experience and the tendency to experience emotional contagion (Cardeña et al., 2008). Furthermore, neuroimaging studies have revealed activation of the mirror neuron system when individuals observe emotional expressions (Decety and Jackson, 2004; Rizzolatti and Craighero, 2004). Facial electromyography studies further show that people mimic facial expressions of happiness or anger within milliseconds of exposure, influencing their own affective states (Dimberg et al., 2000). Social context apparently modulates this process. In particular, Barsade (2002) found that a leader’s emotional expressions significantly influenced emotional tone and performance of the follower-group. Kramer et al. (2014) extended these findings to digital spaces, demonstrating that emotional tone on social media platforms can causally influence users’ mood and content. These phenomena are primarily linked to Mechanism 3: Automatic mimicry and Mechanism 5: Interpersonal entrainment. But the leader and platform effects also highlight Mechanism 4: Attention synchronization as an amplifier of emotional contagion (Mechanism 4 → Mechanism 3 → Mechanism 5).

Parkinson and Simons (2009), Parkinson (2011) explained that emotional contagion may be shaped by affective mimicry or social appraisal. Although individuals tend to automatically mirror others’ emotional expressions—such as facial cues, gestures, or tone—this alone does not fully account for the emotional shifts observed in social contexts. This implies that other psychological processes contribute to the transfer of affect. One such process is social appraisal, which involves interpreting the emotional meaning of another person’s expression within a given context. Rather than simply reacting to the emotion itself, observers evaluate what that emotion reveals about the situation or object of attention. For example, seeing someone display fear or anger may lead the observer to reevaluate their own stance toward a shared environment or stimulus. This interpretive process allows individuals to align their emotional responses based not only on observed behaviors but also on perceived social meaning. Consequently, future research should pay closer attention to how people appraise emotional expressions in relation to contextual cues, particularly focusing on the targets or causes of those emotions. This will help to clarify how emotional responses are socially shaped and transmitted across individuals and groups. This emphasis on interpretation implicates Mechanism 6: Social appraisal and shows that appraisal moderates mimicry-driven affect (Mechanism 6 → Mechanism 3: Automatic mimicry).

To study these ideas and proposals, Doherty (1997) designed the *Emotional Contagion Scale* (ECS) to assess the extent to which individuals experience the transfer or contagion of emotions in social interactions. This a 15-item psychometric instrument measures both the cognitive and affective components of emotional contagion, which include the perception of emotional cues from others and the subsequent emotional response triggered within the individual. The ECS covers the five basic emotions of Happiness, Love, Fear, Anger, and Sadness. Statistical analysis showed that all 15 items load onto a single factor, though the positive and negative emotions show some internal grouping. Psychometric evaluations using Classical Test Theory of the ECS have shown strong reliability, with Cronbach’s alpha coefficients ranging from 0.80 to 0.90, indicating good internal consistency (Doherty, 1997). Furthermore, the ECS has strong construct validity, correlating positively with measures of emotional empathy and social sensitivity. The ECS evidence supports the role of dispositional receptivity in emotional transfer, consistent with Mechanism 1: Suggestibility.

The scale is widely used in social psychology, emotional intelligence research, and interpersonal communication studies. It has been employed to examine the role of emotional contagion in group dynamics, leadership, social bonding, and emotional transmission in organizational or community settings (Hatfield et al., 1993a,b). It also has clinical applications, particularly in the assessment of disorders characterized by emotional dysregulation, such as mood disorders or autism spectrum disorders, where emotional contagion may be altered or impaired (Panksepp and Biven, 2012). Overall, the ECS provides a reliable and valid method for evaluating emotional contagion in both research and clinical settings, offering insights into the ways emotions are transmitted within social contexts.

The Contagion of Affective Phenomena Scales (CAPS) is a newer 21-item psychometric tool designed to measure individual differences in susceptibility to affective contagion across a range of emotions (Clarkson et al., 2025). It aims to capture not only general susceptibility to emotional contagion but also differential sensitivity to six affective states (i.e., a six-factor structure of items), including Anger, Fear, Anxiety, Sadness, Excitement, and Happiness. Therefore, the CAPS provides a multidimensional assessment that expands upon earlier unidimensional models like Doherty’s (1997) ECS. Validation studies suggest that the CAPS has strong internal consistency and construct validity, correlating significantly with related constructs such as empathy, emotional reactivity, and interpersonal sensitivity. Moreover, the CAPS has been shown to predict emotion-driven social behaviors in experimental and naturalistic contexts, suggesting utility for both clinical assessment and research on social affective processes (Clarkson et al., 2025). The CAPS’ multidimensional findings further implicate Mechanism 1: Suggestibility alongside Mechanism 3: Automatic mimicry in explaining individual variation (Mechanism 1 → Mechanism 3).

The CAPS and ECS both assess individual susceptibility to catching emotions from others and thus have a shared interest in interpersonal affective processes. Both instruments also conceptualize emotional contagion as an involuntary, affective response to others’ emotions and employ self-report items to measure this phenomenon. However, the scales diverge in their structure and scope. Whereas ECS operationalizes emotional contagion as a single factor, the CAPS argues for a multidimensional construct. The ECS also primarily reflects emotional mimicry and affective resonance at a general level, as compared to the CAPS which provides more granular insight into emotion-specific vulnerabilities, arguably making it more applicable to targeted research and clinical contexts.

Domain summary: Mechanisms most supported — 1, 3, 5; Mechanisms amplified by context — 4, 6 (see Table 3). These emotional-contagion patterns converge on an input→synchronization pathway in which suggestibility and mimicry (Mechanisms 1–3) initiate affective resonance that shared attention and entrainment (Mechanisms 4–5) consolidate; the model developed below integrates these dynamics into a unified multilevel account.

## 5 Exploring perceptual contagion

Our review suggests that this modality largely concerns Mechanism 2: Expectancy and framing, Mechanism 4: Attention synchronization, and Mechanism 9: Feedback loops and threshold dynamics. Perceptual contagion refers to the socially-driven spread or adoption of discrete sensory experiences, in which an individual’s perceptions are shaped or altered by social cues, group expectations, or suggestive contexts (Wegner, 2002). This phenomenon is exemplified by the notion of placebo and nocebo effects (for reviews, see Frisaldi et al., 2023; Stein et al., 2025b). Placebo analgesia, for instance, involves real reductions in pain perception due to the belief in receiving treatment, underpinned by endogenous opioid and dopaminergic activity (Wager et al., 2004; Benedetti et al., 2005). Conversely, nocebo effects can result in physiological symptoms from negative expectations (Colloca and Miller, 2011). This domain also captures other important phenomena, including the Baader–Meinhof phenomenon, i.e., the frequency illusion when people begin noticing a concept more frequently after first encountering it. This perceptual bias is linked to selective attention and confirmation bias, revealing how perception can be shaped by recent cognitive salience and social reinforcement (van der Meulen, 2022). These expectation-based perceptual shifts are anchored in Mechanism 2: Expectancy and framing and are moderated by Mechanism 1: Suggestibility (Mechanism 2 ← Mechanism 1).

Contemporary theories of perception, such as predictive coding and Bayesian brain models, reinforce this view by conceptualizing perception as an inferential process. According to these frameworks, the brain constantly generates predictions about sensory input based on prior experiences and updates these models in response to incoming data (Friston, 2005; Hohwy, 2013). In this context, contagion occurs when group norms, language, or cues bias individuals’ priors, causing them to perceive phenomena that align with collective expectations. This explains how simple verbal suggestion or media exposure can lead to the perception of unusual bodily sensations, phantom odors, or ambiguous threats—especially in uncertain or stressful environments. Predictive coding arguably frames perceptual contagion as a function of biased priors, i.e., Mechanism 2: Expectancy and framing, operating through attention (Mechanism 4) and feedback dynamics (Mechanism 9).

Beyond analog, real-world settings, perceptual contagion is increasingly mediated and amplified by digital technologies (addressed in more detail in a later section). Repetitive exposure to emotionally charged imagery, conspiracy narratives, or hyperrealistic content (e.g., deepfakes) can recalibrate individuals’ perceptual thresholds. For instance, social media algorithms often prioritize novel, dramatic, or emotionally salient content, reinforcing selective attention and making certain visual or auditory patterns appear more frequent or meaningful than they are (Brady et al., 2020). This can lead to perceptual saturation, confirmation biases, and even false memory formation, especially in ideologically homogenous online spaces (cf. Krockow et al., 2023). In extreme cases, repeated exposure to misleading or anxiety-provoking stimuli may contribute to perceptual derealization, hypervigilance, or collective misinterpretation of benign stimuli as threats. Repetition and algorithmic salience implicate Mechanism 4: Attention synchronization and show how perceptual priors are externally reinforced (Mechanism 4 → Mechanism 2).

Neuroscientific research further supports the role of top-down modulation in perceptual contagion. Functional neuroimaging studies have identified specific brain regions that mediate expectation-based perceptual changes. For example, the anterior cingulate cortex and anterior insula are associated with interoceptive awareness and the integration of affective states into sensory experience, while the default mode network is implicated in self-referential thinking and the incorporation of belief into perception (Petrovic et al., 2002; Wager et al., 2004; Barrett and Simmons, 2015). These findings suggest that contagious perceptions—whether positive (e.g., placebo) or negative (e.g., nocebo)—are not hallucinatory anomalies but rather predictive misalignments between expectation and sensory input. These neurobiological correlates further map onto Mechanism 2: Expectancy and framing and Mechanism 5: Interpersonal entrainment when social signals tune interoceptive inference.

Furthermore, perceptual contagion is not limited to generalized or abstract experiences; it often manifests in modality-specific forms, particularly in visual and auditory domains. Visual contagion may include shared sightings of anomalous lights, patterns, or figures (e.g., during religious rituals or paranormal events), while auditory contagion can involve group experiences of phantom sounds, indistinct voices, or environmental anomalies (Houran and Lange, 1996; Lange and Houran, 1997, 2001a). We discuss these ideas in more depth in a later section devoted to anomalous experiences, but the above findings collectively suggest that perceptual contagion is not merely about suggestion or belief—it instead reflects a complex interaction between neural prediction, environmental context, social influence, and sensory ambiguity. From both a cognitive and neurobiological standpoint, perception is a fluid, dynamic process that is constantly shaped by internal models and external cues. These insights challenge traditional dichotomies between “real” and “illusory” perception and underscore the power of social environments—both physical and digital—in shaping what people experience and how they interpret it. Modality-specific contagion exemplifies how Mechanism 2 and Mechanism 4 combine with situational ambiguity to produce perceptual convergence (Mechanism 2 → Mechanism 4 → Mechanism 9).

Domain summary: Mechanisms most supported — 2, 4; Mechanisms interacting/moderating — 1, 5, 9 (see Table 3). These perceptual findings align with an expectancy-driven input stage amplified by attention synchrony and feedback dynamics (Mechanisms 2, 4, 9), producing shared perceptual priors that the model below situates at the interface between individual and large-scale processes.

## 6 Exploring behavioral contagion

Our review suggests that this modality emphasizes Mechanism 3: Mimicry, Mechanism 7: Network and institutional amplifiers, Mechanism 8: Moralization and affective intensity, and Mechanism 9: Threshold dynamics. This final category refers to the propagation of actions across individuals, often without conscious awareness or rational deliberation (Wheeler, 1966). This phenomenon has interested observers of crowd behavior for centuries (Mackay, 2011). Common examples include contagious yawning, laughter, and posture mimicry (Wheeler, 1966; Provine, 1986, 1992). However, this phenomenon also encompasses more complex and consequential behaviors—such as crowd dynamics, protest escalation, online trolling, health-related actions like mask-wearing or panic buying, and even historical events like the dancing plague, a peculiar public health conundrum involving collective motor behaviors (Donaldson et al., 1997). Several different mechanisms potentially mediate or moderate such effects, ranging from automatic imitation to social learning and neural entrainment. These automatic actions are principally expressions of Mechanism 3: Automatic mimicry and are facilitated by baseline receptivity (Mechanism 1).

A foundational explanation comes from Bandura’s (2001) social cognitive theory, which proposes that people acquire and perform behaviors through observational learning, particularly when behaviors are modeled by others perceived as competent or socially rewarded. This involves not only mimicry but also vicarious reinforcement, where witnessing others being rewarded or punished for certain behaviors influences the observer’s likelihood of imitation. Such modeling processes are especially potent in social groups or hierarchies where prestige bias—the tendency to imitate high-status individuals—plays a significant role in behavioral diffusion (Henrich and Gil-White, 2001). In parallel, mimetic theory (Girard, 1987) suggested that people often desire things because others desire them, a phenomenon known as mimetic desire. This theory highlights how behavioral contagion is not always rational or functional; instead, imitation is sometimes driven by subconscious rivalry or identity-seeking, particularly in contexts where group affiliation or symbolic capital is at stake. These dynamics help to explain phenomena such as viral consumer trends, collective outrage, or social media challenges. Observational learning and prestige effects show the centrality of Mechanism 7: Network and institutional amplifiers together with Mechanism 3 (Mechanism 7 → Mechanism 3).

Adding to these cognitive and motivational mechanisms is a growing body of evidence from social neuroscience that demonstrates how behavioral contagion may relate to inter-brain synchronization. This phenomenon—also known as inter-brain phase synchronization—refers to the temporal alignment of neural activity across two or more individuals during joint tasks or social interaction. This alignment often occurs in the timing, phase, frequency, or amplitude of neural oscillations and is typically studied using hyperscanning techniques like electroencephalography (EEG) or functional near-infrared spectroscopy (fNIRS) (Dumas et al., 2010; Czeszumski et al., 2020). Such synchronization has been observed across various social contexts, including conversational turn-taking, musical collaboration, dance, teacher-student interactions, romantic relationships, and team-based problem-solving (Hasson et al., 2012; Liu et al., 2016; Zheng et al., 2020). These findings on neural synchrony support Mechanism 5: Interpersonal entrainment as a substrate for coordinated behavior.

The mechanisms underlying inter-brain synchronization are thought to include shared sensory inputs, mutual prediction, and active interpersonal attunement. While some researchers argue that these findings represent genuine neural coupling between individuals, others caution that the effects may instead reflect shared environmental inputs rather than direct inter-brain resonance (Burgess, 2013). Nonetheless, inter-brain synchrony has been associated with increased cooperation, empathy, and mutual understanding, making it highly relevant to fields like education, psychotherapy, and human-computer interaction (Konvalinka and Roepstorff, 2012; Pan et al., 2020). These findings suggest that cognition during social interaction may be partially distributed across individuals, rather than isolated within individual brains.

While mimicry-based emotional contagion and shared neural dynamics help to explain how behaviors and emotions spread, they do not fully account for the more nuanced, group-level coordination observed during communal activities. Parkinson (2020) suggested that emotional convergence (or resonance) within groups also can emerge through dynamic calibration. This is where individuals engaged in shared tasks co-regulate their focus, actions, and emotional responses in real-time. This process often involves mutual “entrainment,” facilitated by interaction rituals like synchronized singing or dancing that help to align group members’ attention and affective states. Such alignment goes beyond mimicry or appraisal, offering a richer account of how collective emotional experiences arise during joint activities. Entrainment in rituals implicates Mechanism 5 and demonstrates how interpersonal synchronization elevates mimicry into coordinated group action (Mechanism 5 → Mechanism 3 → Mechanism 9).

Behavioral contagion also operates through social conformity and demand characteristics. Demand characteristics refer to subtle cues in an experimental setting that influence participants’ behavior based on their perceptions of the researcher’s expectations (Orne, 1962). These cues can lead to changes in behavior that reflect compliance rather than genuine responses, threatening internal validity. Orne’s (1962) foundational work argued that much of what participants do in experiments may be shaped not by the variables being studied, but by their assumptions about what is expected of them. Subsequent research has elaborated on this framework, showing that demand characteristics can emerge through experimental instructions, task framing, or the physical presence of the experimenter (Nichols and Maner, 2008). Weber and Cook (1972) long ago developed the concept of the “good subject effect,” where participants act in ways that they believe will confirm the hypothesis. Efforts to mitigate demand characteristics include the use of deception, double-blind procedures, and post-experiment questionnaires to detect suspicion (McCambridge et al., 2012). Yet, concerns persist in fields such as social psychology and behavioral research, where constructs are often susceptible to social desirability and expectancy effects (Rosenthal, 1966). These topics have resurged due to recent research (Arnull et al., 2024; Guenole et al., 2024; Lange et al., in press) that strongly suggests the results of standard factor analysis point to a shared conception of social reality (cf. Wittgenstein, 1953/1958), rather than to the nature and structure of actual human traits. These methodological and normative pressures imply that Mechanism 6: Social appraisal and Mechanism 1: Suggestibility jointly shape observed behavioral conformity (Mechanism 6 ← Mechanism 1).

Relatedly, group conformity effects refer to the influence of group norms and pressure on individual behavior, even in the absence of explicit coercion. Asch’s (1951) seminal experiments demonstrated that individuals often conform to a majority opinion, even when it is objectively incorrect. This has been interpreted as evidence for both *normative* (desire for acceptance) and *informational* (belief the group is better informed) social influence (Deutsch and Gerard, 1955). Later work has extended this paradigm to more complex and ecologically valid contexts. Crutchfield (1955) used a more anonymous setting and still observed significant conformity effects, suggesting that overt peer pressure is not necessary for group norms to shape individual responses. Neuroscientific studies have supported these findings, indicating that conformity is associated with activation in brain regions involved in conflict monitoring and rewards (Klucharev et al., 2009). Moreover, social conformity can be amplified in ambiguous or stressful situations, or when individuals are uncertain about their judgments (Baron et al., 1996).

Although often studied separately, demand characteristics and group conformity effects share a common underlying mechanism: social cues that influence behavior in ways not directly tied to the independent variables. Researchers have noted that group settings may heighten susceptibility to demand characteristics due to increased sensitivity to social norms (Goffman, 1959; Turner, 1991). For example, participants may conform to perceived experimental norms not only to align with the group but also to fulfill their role as a “good participant.” This blending of demand characteristics and conformity pressures complicates interpretation, especially in group-based or field experiments. Methodologically, controlling for these combined effects requires careful experimental design, such as the inclusion of control groups unaware of the study’s aims, or the use of implicit measures that reduce participants’ ability to infer expectations (Kazdin, 2016).

In the digital age, behavioral contagion is further magnified by algorithmic amplification and the architecture of social media. Online behaviors—such as sharing posts, participating in viral trends, or engaging in moral outrage—are shaped by metrics like likes, retweets, and trending topics, which function as behavioral cues reinforcing conformity and visibility (Berger and Milkman, 2012). Social media platforms like TikTok and YouTube accelerate the spread of behaviors through memetic transmission, where symbolic actions or gestures are replicated, remixed, and recirculated at scale. This architecture also facilitates unintended amplification, as seen in the Streisand effect, where attempts to suppress content only increase its virality (Zuckerman, 2009). Online architectures ostensibly convert local imitation into large-scale behavioral spread via Mechanism 7: Network and institutional amplifiers and accompanying threshold dynamics (Mechanism 7 → Mechanism 9).

There are sometimes dark manifestations of such contagion. For instance, Christakis and Fowler (2007) reported that obesity appears to spread through social networks, with individuals more likely to become obese if their friends, siblings, or spouses do, suggesting that social connections significantly influence weight gain. Research into media contagion also has found that reports of suicide, especially among adolescents, can increase suicide rates through imitation (Gould et al., 2003; Martínez et al., 2023). Towers et al. (2015) identified similar dynamics in mass shootings, proposing that media coverage and notoriety may serve as behavioral cues for susceptible individuals. Importantly, not all behavioral contagion is negative. Research also demonstrates that prosocial behaviors—including helping, donating, and health-promoting actions—spread across social networks. For example, Christakis and Fowler (2013) showed that behaviors like quitting smoking, exercising, or expressing happiness tend to cluster within social groups due to peer influence and emotional resonance. Similarly, Aral and Nicolaides (2017) found that exposure to peers’ physical activity patterns on social platforms positively influenced users’ own behaviors. During public health crises, behaviors like mask-wearing or handwashing are often reinforced through social modeling and policy visibility, rather than individual conviction alone (Singhal and Rogers, 2003; Goldstein et al., 2008). These mixed outcomes highlight Mechanism 7 and Mechanism 8: Moralization and affective intensity as key determinants of whether behavioral contagion produces prosocial or harmful cascades (Mechanism 7 + Mechanism 8 → Mechanism 9).

Mass psychogenic illness (MPI) —sometimes called “collective anxiety attacks” (Bartholomew and Victor, 2004, p. 229) —is a phenomenon involving the rapid spread of illness signs or symptoms within a cohesive group, with no identifiable organic cause (Colligan and Murphy, 1979; Colligan et al., 1982; Bartholomew and Wessely, 2002). François Sirois (1974) proposed a widely cited diagnostic paradigm for identifying MPI or what he termed episodes of epidemic hysteria. His framework helps to distinguish such outbreaks from those caused by biological agents or environmental toxins by outlining a set of characteristic features. These include (a) the absence of a plausible organic basis for symptoms, despite thorough medical testing; (b) the rapid spread of symptoms among individuals in close proximity, often through visual or verbal contact; (c) a predominance of anxiety-related symptoms, such as fainting, hyperventilation, or dizziness; (d) a high rate of recovery without the need for medical intervention; (e) a greater prevalence among females, particularly adolescents or young adults; (f) the presence of a triggering event (e.g., a strange odor, stress, or panic) that initiates the episode; and (g) a social contagion mechanism, whereby symptoms spread through suggestion or imitation rather than through exposure to a biological pathogen (for a further review and discussion, see Page et al., 2010).

Sirois (1974) also noted that such episodes typically involve a high rate of recovery without medical intervention, disproportionately affect females—especially adolescents or young adults—and often follow a triggering event such as a noxious odor or stressful incident. Importantly, the spread of symptoms tends to occur through suggestion or imitation, rather than through a physical contagion mechanism. The spread of symptoms occurs through social pathways: visual exposure, verbal suggestion, and modeling. Individuals who watched peers collapse often reported similar sensations, and suggestion could directly trigger subsequent episodes (Sapkota et al., 2014). These mechanisms reveal that psychological states are transmitted interpersonally, with symptom expression serving as both a communicative act and a trigger for further spread. Subsequent research has elaborated on and supported Sirois’ model. For example, Boss (1997) reviewed numerous cases of mass psychogenic illness and affirmed the utility of Sirois’ criteria for public health surveillance and response. Similarly, Bartholomew and colleagues applied this paradigm to historical and contemporary outbreaks, emphasizing its value in preventing unnecessary medical or logistical interventions in cases where psychosocial mechanisms are at play (e.g., Bartholomew and Sirois, 1996; Bartholomew and Rickard, 2014; Bartholomew and Baloh, 2020).

Recent research has further expanded our understanding of MPI by exploring its occurrence across diverse cultural settings through psychosocial lenses (e.g., Penna, 2019; Yan, 2023). One case-control study in Nepal demonstrated that adolescents affected by MPI outbreaks exhibited a clear profile of pre-existing vulnerability, including significantly higher suggestibility and dissociative tendencies compared to their unaffected peers (Sapkota et al., 2020). Symptoms typically include headache, dizziness, fainting, nausea, and hyperventilation—often mimicking genuine physical illness but occurring in the absence of any physical pathogen (Jones, 2000). A notable example of MPI, cited by Pradhan et al. (2024), occurred at a girls’ boarding school in central Odisha. This involved a 12-year-old student (Miss A), who experienced sudden episodes of fainting, abdominal pain, and convulsions. Her symptoms rapidly spread to nearly 100 peers. Subsequent medical examinations revealed no physiological trigger. Correspondingly, the investigators attributed the outbreak to psychogenic factors, influenced by cultural narratives involving malevolent spirits. Such events can be understood as culturally-shaped idioms of distress, where psychosocial problems and suffering is expressed through somatic and dissociative symptoms that are legible within the local cultural framework (Sapkota et al., 2014). This event highlights the role of cultural context and social dynamics in amplifying emotional and perceptual contagion, particularly among young, impressionable individuals in high-stress environments.

MPI is most commonly observed in environments characterized by close social interaction, such as schools, factories, or religious communities (Boss, 1997; for reviews and case studies, see Bartholomew and Sirois, 1996; Bartholomew and Rickard, 2014; Bartholomew and Baloh, 2020) and is generally classified into two types: *anxiety-based* MPI, which involves transient and acute symptoms like fainting or nausea, and *motor-based* MPI, which features longer-lasting and more unusual motor symptoms such as twitching or convulsions (Wessely, 1987). Episodes often begin with an index case, typically someone who is high-status or emotionally expressive and spread rapidly through visual or verbal contact (Small and Nicholi, 1997).

Epidemiologically, MPI disproportionately affects females and younger populations, especially adolescents (Colligan et al., 1982; Sapkota et al., 2020). This gender disparity has been linked to culturally-mediated stressors and inequalities that disproportionately affect women and girls, shaping their expression of psychological distress (Sapkota et al., 2014, 2019). While the exact mechanism is debated, emotional distress, social suggestibility, and the modeling of symptoms are commonly implicated (Bartholomew and Sirois, 1996). A path analytic study suggested that for adolescents, the pathway to dissociative experiences—a core feature of many MPI outbreaks—is often mediated by factors such as higher psychosocial distress and cognitive and personality factors (i.e., susceptibility to cognitive failures, emotional contagion, fantasy proneness, etc.) (Sapkota et al., 2019).

Cultural context also plays a crucial role, shaping both the content of symptoms and the social response to the outbreak (van der Meulen, 2022). Outbreaks of MPI and possession are not experienced as arbitrary fainting spells but rather interpreted through culturally resonant idioms of distress. In Nepal, collapses were framed as “witchcraft” or “spirit possession,” which amplified their credibility and heightened community concern. Such framings often reflected beliefs that spirits punish individuals for past-life misdeeds, the wrongdoings of family members, curses directed at the afflicted, or simply the misfortune of crossing a spirit’s path (Sapkota et al., 2014). This demonstrates how cultural narratives and explanatory models provide a script that makes contagion-like experiences intelligible, expectable, and more likely to spread.

MPI is not feigned or consciously produced; rather, it arises involuntarily and often resolves quickly once the group disperses or the perceived threat is de-escalated. This distinguishes it from malingering or factitious disorder (Jones, 2000). Recent studies also have explored MPI in the context of digital environments, suggesting the potential for “virtual” mass psychogenic events spread via social media (Bartholomew and Baloh, 2020). Such cases raise questions about the boundaries between traditional, face-to-face contagion and technologically mediated psychosocial influence. These studies underscore that MPI, and related contagion phenomena, emerge from the interplay of psychological vulnerability, cultural framing, and social transmission mechanisms. This integrated view helps to explain why outbreaks recur in school and community settings worldwide and why symptom expression so often reflects the local cultural repertoire of distress.

Contemporary neurobiological and psychiatric research suggests that MPI lies at the intersection of conversion disorder, social anxiety, and mass suggestibility, though it remains distinct from malingering or factitious disorder (Peters, 2001). Treatment is best approached through public reassurance, rapid identification of the psychosocial trigger(s), and containment of media coverage, rather than through medicalization or stigmatization (Wessely, 1987; Boss, 1997). Modern outbreaks often receive media attention, which can amplify symptom spread via additional PC (Ali-Gombe et al., 1996). The 2011–2012 outbreak in Le Roy, New York, illustrated this dynamic: intense media coverage and competing scientific explanations magnified both the reach of symptoms and public anxiety. Anthropological analysis has shown how neurological experts framed the illness as “psychogenic,” thereby sidelining environmental concerns and reinforcing stigma (Goldstein and Hall, 2015), whereas public health scholarship highlights how poor communication and the pejorative “mass hysteria” label deepened mistrust and fueled controversy (Bartholomew, 2016). Other scholars have proposed that MPI represents a form of collective stress reaction to sociocultural pressures, workplace dissatisfaction, or traumatic change (Bartholomew and Wessely, 2002; Mawson, 2007).

Finally, there are PC-related phenomena involving coordinated group behaviors. In particular, *mob behavior* (i.e., a group of people acting together in an emotional, often aggressive or irrational way, usually influenced by the crowd rather than individual thinking) and *hooliganism* (i.e., rowdy, violent, or destructive behavior, often linked to sports fans or public disturbances) are now both widely understood as identity-driven, emotionally charged forms of collective action rather than merely irrational crowd phenomena. Early contagion theories (e.g., Le Bon, 2002) depicted such behavior as mindless and automatic, but contemporary models—especially the social identity approach—highlight the role of shared group norms, intergroup dynamics, and coordinated actions (Reicher, 1987; Drury and Reicher, 2000). In contexts such as football hooliganism, violence is often ritualized and symbolic, expressing group loyalty, masculine identity, and territorial defense (Dunning et al., 1988; Spaaij, 2008).

PC particularly manifested as the rapid spread of emotions through mimicry and group identification (Hatfield et al., 1993a,b), nevertheless remains central as a mechanism that amplifies collective arousal and aligns individuals with emergent group norms. In the context of mob behavior and hooliganism, emotional contagion can escalate group arousal (Berger, 2011), leading to a feedback loop in which individual restraint gives way to collective enactment. Neuroscientific evidence supports this, showing that mirror neuron systems and emotional mimicry facilitate the rapid spread of affective states within groups (Gallese and Goldman, 1998). Emotional contagion may not cause mob behavior per se, but it plays a vital role in amplifying group norms and sustaining coordinated collective action, especially in high-intensity, intergroup contexts such as sports riots and protest violence (Drury et al., 2009).

Domain summary: Mechanisms most supported — 3, 5, 7; Mechanisms moderating direction/intensity — 1, 6, 8, 9 (see Table 3). These behavioral patterns indicate that automatic imitation and prestige-based diffusion (Mechanisms 3 and 7), when coupled with moralization and threshold dynamics (Mechanisms 8–9), convert local imitation into population-level cascades in the multilevel model developed below.

## 7 Role of contagion in modern controversies

In contemporary society, PC is accelerated by digital platforms and mass media; this section examines how contagion dynamics underlie health scares and panic, vaccine and other misinformation, conspiratorial belief formation and polarization, trolling and intentional provocation, reactive amplification (Streisand-type effects), memetic diffusion of behaviors, digitally mediated mass psychogenic events and sick-building complaints, and controversial phenomena such as Rapid-Onset Gender Dysphoria—showing how PC modalities interact with algorithmic salience, prestige signaling, moralization, and feedback loops to produce rapid, persistent, and sometimes harmful collective outcomes.

### 7.1 Health scares and mass panic

Events like viral outbreaks, environmental hazards, or vaccine misinformation exemplify how emotional contagion can rapidly escalate into mass panic or irrational behavior. The H1N1 pandemic, Ebola outbreaks, and most recently, the COVID-19 crisis have shown how public fear, amplified by media channels, can trigger widespread anxiety, hypervigilance, and even panic buying (Cottingham, 2024). Studies of emotional contagion underscore that *fear*—when propagated through social networks—can spread rapidly, with individuals becoming highly suggestible to exaggerated or inaccurate information about health risks (Van Bavel et al., 2020).

In the case of vaccine misinformation, studies have demonstrated that emotional appeals, particularly fear-based messaging, can facilitate the spread of anti-vaccine sentiment across social media platforms, despite the absence of scientific backing (Kata, 2012). Misinformation about vaccine side effects, such as the now-debunked link between the MMR (measles) vaccine and autism (Taylor et al., 2014), spreads primarily through emotional contagion—where fear, distrust, and heightened emotional responses influence beliefs about medical interventions, irrespective of factual evidence (Kata, 2012; Hornsey et al., 2018). This dynamic not only compromises public health but also exposes the vulnerability of collective decision-making in an emotionally charged digital environment.

### 7.2 Conspiratorial thinking and group polarization

Conspiratorial thinking, which involves belief in hidden, malevolent forces guiding events, is another domain in which PC ostensibly operates on a large scale. The spread of modern-day conspiracy theories—such as the “Trump-Russiagate Collusion” hoax (Boyd-Barrett and Marmura, 2023), so-called “chem trails” allegedly involving the government secretly spraying harmful or mysterious substances via airplanes (Tingley and Wagner, 2017), or misinformation about the efficacy of COVID-19 vaccines (Wu et al., 2023) —illustrates how perceptual contagion and expectancy effects can lead to the adoption of increasingly extreme beliefs (pro or con towards a topic) within certain social groups (Goertzel, 1994; Douglas et al., 2017). These theories often originate from small, fringe communities but gain traction as they are amplified through social media algorithms, creating “echo chambers” where groupthink and selective reinforcement foster the proliferation of attitudes and beliefs without exposure to contradictory information (Cinelli et al., 2021).

Conspiracy theories are often fueled by emotional contagion, particularly suspicion and distrust, which become viral when framed by charismatic leaders, media figures, or even ordinary social media influencers. Once an individual expresses a conspiratorial belief, others within the group are inclined to mimic those thoughts, not based on rational evidence but through social conformity and emotional resonance (van der Linden, 2015). This collective reinforcement of paranoid worldviews not only sustains conspiracy movements but also magnifies their effects, as individuals are drawn further into these belief systems through contagion dynamics.

Furthermore, group polarization, a process whereby discussions within a group lead to more extreme positions (Sunstein, 2009), plays a significant role in the spread of conspiratorial thinking. As individuals with shared conspiratorial beliefs interact and reinforce each other’s suspicions, their beliefs become more extreme and detached from reality, creating a “feedback loop” that strengthens the emotional contagion and motivates further belief entrenchment.

### 7.3 Trolling and digital disinhibition

The phenomenon of trolling—where individuals intentionally provoke or disrupt online communities through inflammatory, misleading, or offensive comments—can also be understood through a PC lens. Behavioral contagion plays a critical role in the escalation of trolling behavior, as individuals often engage in trolling in response to others’ provocative comments, creating a snowball effect that amplifies hostility and aggression in online spaces (Suler, 2004). The anonymity afforded by the internet allows individuals to bypass social norms, and the digital disinhibition effect enables individuals to express extreme opinions or engage in disruptive behaviors that they might otherwise avoid in face-to-face interactions (Joinson, 2007).

Emotional contagion also operates within online trolling environments, as participants’ emotional states—such as anger, frustration, or amusement—are transferred across social media platforms. Trolling not only affects the emotional state of the target but can also trigger a cascade of emotional responses within a broader community, fostering a hostile or defensive atmosphere (Wiseman et al., 2003). This contagious negativity leads to an escalation of conflict, where users continually mirror each other’s emotional tones, further distorting the social dynamics and often disrupting the functionality of online communities (Lu and Hong, 2022).

Additionally, trolling behavior can be seen as a strategic form of PC, where individuals or organized groups intentionally provoke emotional reactions to advance political, social, or ideological agendas. The spread of anger, mistrust, or disbelief through viral trolling campaigns amplifies societal divisions, contributing to polarization and the entrenchment of opposing viewpoints (Simchon et al., 2022).

### 7.4 Social media influence: the Streisand effect and viral misinformation

A significant modern example of PC is the Streisand Effect, which occurs when attempts to suppress information inadvertently draw more attention to it, often leading to its viral spread. Named after Barbra Streisand’s legal attempt to remove photographs of her Malibu estate from the internet, this phenomenon illustrates how reactive suppression can become a form of behavioral contagion, where the action to suppress information amplifies its visibility across social media platforms (Zuckerman, 2009). The Streisand Effect is a direct manifestation of social amplification, where individuals and groups are increasingly motivated to engage with suppressed or censored content due to its perceived importance or the drama of the suppression itself.

This effect is closely tied to viral misinformation, where the emotional charge of a suppressed narrative or controversial topic sparks intense engagement and further dissemination through social networks. As more individuals amplify their own emotional reactions—whether outrage, humor, or disbelief—a contagious loop of attention and discussion is formed, significantly increasing the likelihood that misinformation, once obscured, will rapidly circulate (Vosoughi et al., 2018).

### 7.5 Mass hysteria, sick building syndrome, and cultural contagion

MPI continues to be one of the most striking examples of PC. Large groups of individuals exhibit similar, often inexplicable physical symptoms in the absence of a medical cause, suggesting that perceptual contagion and suggestion can lead to widespread collective psychogenic experiences. Classic examples include episodes like the Salem witch trials, the dancing plague of 1518, and more contemporary occurrences such as the 1998 school gas scare in the United States (Bartholomew and Wessely, 2002). Other modern cases have been interpreted in terms of paranormal agencies (e.g., Chen et al., 2003; Sapkota et al., 2020), with some authors even proposing the specific notion of “paranormal contagion” (Ritson, 2021; McCue, 2022). Regardless, MPI episodes typically involve emotional and social contagion, with fear and anxiety triggering behaviors in highly suggestible groups (Colligan et al., 1982).

Recent cases of mass hysteria in schools or workplaces demonstrate how the emotional contagion of fear, coupled with social pressure, can induce a collective belief in illness or danger, even when apparently no physical threat exists (Goetz, 2000). These instances emphasize how cultural narratives—fear of contamination, illness, or supernatural intervention—interact with contagion dynamics to form shared, but often irrational, group behaviors. In these contexts, social media plays a role in amplifying the contagion process, as anxious posts and viral warnings can escalate fears even further, creating a feedback loop where individuals’ anxieties are perpetuated and shared across wider networks.

Sick building syndrome (SBS) involves nonspecific symptoms such as headaches, eye irritation, and fatigue, often attributed to time spent in a particular building without identifiable environmental causes. Some research implicates the role of poor ventilation in these cases (e.g., Hedge et al., 1989; Hedge and Erickson, 1998; Lu et al., 2018), but most studies underscore the importance of individual differences and psychological variables, irrespective of the presence of environmental issues. Recent research indeed suggests that psychosocial factors, and PC effects play a significant role in SBS, whereby individuals in a group may begin to report similar health complaints due to shared beliefs or anxieties even in the absence of environmental triggers (Bartholomew and Wessely, 2002). MPI research therefore highlights the influence of psychosocial influences in the spread of symptoms, with triggers like rumors or odors exacerbating collective symptom reporting (Hedge and Erickson, 1998). Additionally, psychological stressors, such as workplace stress and poor organizational climate, often contribute to the prevalence of SBS symptoms. For instance, studies indicate that high stress and low cooperation among colleagues are associated with increased symptom reporting (Ooi and Goh, 1997). Therefore, effective SBS management requires addressing both physical and psychological factors, including improving air quality and fostering supportive organizational environments to mitigate the PC effects (for an overview, see Nag, 2019).

### 7.6 Rapid-onset gender dysphoria (ROGD)

Some clinicians and scholars (e.g., Littman, 2018) have proposed that ROGD, particularly among adolescent girls and in peer clusters, may reflect a socially-mediated process, rather than arising solely from individual, long-standing gender incongruence. In this framework, the sudden identification as transgender is seen not as consciously deceptive or malicious, but as emerging through suggestibility, peer influence, and social reinforcement—elements often central to MPI. Because evidence for ROGD is limited and contested, we treat the analogy as a provisional, ethically sensitive hypothesis for further research, not as a clinical claim.

Key parallels with MPI proposed in this view include: (a) *Clustering*: like classical MPI, reports of sudden transgender identification have sometimes appeared in peer groups or schools (Littman, 2018); (b) *Psychological stress*: adolescents experiencing anxiety, depression, or trauma—known risk factors for MPI—may also be overrepresented in ROGD samples (Kaltiala-Heino et al., 2018); (c) *Social modeling and media influence*: MPI often spreads through visual/verbal transmission. In a digital age, social media (e.g., TikTok, Reddit) may serve as a vector for gender identity exploration and reinforcement, analogous to how media has amplified past MPI outbreaks (Bartholomew and Wessely, 2002); (d) *Suggestibility and identity seeking*: adolescents in transitional, uncertain phases may be particularly susceptible to social identification processes, which can mimic contagion dynamics; and (e) *Medicalization*: both MPI and ROGD critiques point to the role of healthcare systems in legitimizing and institutionalizing rapidly emerging, socially influenced symptoms.

However, the analogy between gender identity expression and MPI is highly contested on several grounds. First, the empirical foundation for ROGD is restricted and controversial; ROGD is not recognized as a clinical diagnosis in either the *DSM-5* or *ICD-11*, and its empirical support remains preliminary and widely debated. We therefore refer readers to discussions (e.g., Ashley, 2020; Hutchinson et al., 2020) on important ethical cautions that readers should know about when exploring or studying this controversial hypothesis. Second, classical MPI is typically characterized by acute physical symptoms such as fainting, nausea, or motor disturbances, which differ significantly from the neurological and phenomenological profile of gender identity expression. Third, many critics contend that framing transgender identity through the lens of MPI risks pathologizing legitimate experiences of gender dysphoria and may cause harm to vulnerable youth (Ashley, 2020). Finally, alternative explanations for the rise in transgender identification include increased social acceptance, more precise language for self-understanding, and reduced stigma—none of which imply underlying psychopathology.

Acknowledging the strong sociopolitical and academic sensitivities currently around this topic, we make no firm statements about the ultimate etiology of gender dysphoria or its reportedly elevated prevalence rates over recent years. However, we defend the appropriateness of a “contagion” interpretation whether or not putative ROGD—simply stated—is mostly fueled by (a) increased social awareness and acceptance that motivates individuals with sincerely reported perceptions or beliefs to publicly self-identify as transgender without fear of ridicule or rejection, or (b) behavioral mimicry rooted in peer pressure, cultural currency, or other social forces.

### 7.7 Digital influences: memetic behavior, echo chambers, and algorithmic amplification

The rise of memetic behavior—the shareability or rapid replication and spread of ideas or actions across social networks—illustrates how emotional, perceptual, and behavioral contagion now interact at scale (Berger and Milkman, 2012; Wiggins and Bowers, 2015; Hill et al., 2018). Memes and other viral content circulate with extraordinary speed and evolve as they propagate (Nahon and Hemsley, 2013; Shifman, 2013; Highfield, 2016), and their virality often depends on affective hooks that trigger sharing. Repeated exposure to such content both biases *perception* (making cues more salient) and cues *behavioral imitation* (resharing or enacting), so perceptual and behavioral contagion become tightly coupled in digitally mediated diffusion.

This coupling is intensified by algorithmic curation and engineered engagement metrics: recommendation systems prioritize content that maximizes clicks, dwell time, and emotional reactions, thereby amplifying items that trigger contagion processes irrespective of veracity (Kramer et al., 2014; Xu, 2022; Metzler and Garcia, 2024). Algorithmic salience can elevate peripheral offline material to hyper-visible status online, increasing its likelihood of perceptual uptake and behavioral replication (Berger and Milkman, 2012).

Echo chambers and filter bubbles convert algorithmic exposure into sustained social reinforcement. By preferentially delivering concordant content, platforms reduce cross-cutting information and create feedback loops that intensify beliefs and polarize groups (Sunstein, 2009; Cinelli et al., 2021). In these closed circuits, emotional contagion—outrage, fear, humor, or pride—fuels sharing (Hatfield et al., 1993a,b; Kramer et al., 2014); repeated perceptual cues normalize fringe narratives; and behavioral norms (liking, reposting, participating in challenges) consolidate group identity and action, producing rapid, self-reinforcing cascades (Brady et al., 2020; Fraser, 2020).

The socio-technical coupling has tangible public-health and safety consequences. Algorithmic amplification can escalate harmful behaviors—from copycat violence and livestreamed assaults to the spread of self-harm narratives—by increasing exposure, normalizing extreme acts, and desensitizing audiences (Mrug et al., 2015; Rios and Ferguson, 2020; Milli et al., 2025; Science, Innovation and Technology Committee, 2025). Recording and livestreaming of violence before moderation can encourage imitation and extremism (Kelley and Miles-Novelo, 2025). Suicide contagion exemplifies this risk, as dramatized coverage and repeated exposure can precipitate imitation among vulnerable individuals, and recommendation engines can inadvertently channel at-risk users toward reinforcing content (Luxton et al., 2012; O’Dea et al., 2015; Mueller and Abrutyn, 2024; Spittal et al., 2025).

These contagion effects do not disseminate randomly but follow engineered rules of amplification, i.e., once high-arousal content gains momentum, systems personalize and reinforce its reach across networks, accelerating visibility and persistence (Metzler and Garcia, 2024). Consequently, isolated offline incidents can be transmuted into large-scale online patterns that convert individual vulnerabilities into collective risks. Mitigating these harms requires interdisciplinary responses—platform design changes, public-health interventions, and social-psychological countermeasures—while recognizing that research and policy must keep pace with rapidly evolving technologies (Goldenberg and Gross, 2020; Shelby et al., 2023).

### 7.8 Political polarization and the role of group identity

Political polarization is an area where emotional contagion and social contagion intersect powerfully. The intensification of political divisions, particularly in the United States and other democracies, has been fueled by both traditional and social media channels, where emotionally charged political rhetoric encourages people to adopt more extreme positions (Iyengar et al., 2019). PC dynamics play a central role in this process. As individuals are exposed to emotionally charged political content, they mirror the emotions of others in their social network, contributing to increasingly extreme political positions.

This polarization process can be framed via group-oriented contagion, where individuals within politically homogeneous groups are more likely to adopt the views and emotional states of their peers. Social identity theory (Tajfel and Turner, 1986) explains how individuals’ identities become tied to their political groups, and as they engage with social media content aligned with their beliefs, they experience emotional contagion that reinforces their group identity and biases. Echo chambers on social media amplify this, leading to the radicalization of political views, as users are only exposed to like-minded individuals, creating a vicious cycle of escalating polarization (Benkler et al., 2018).

### 7.9 Terrorism and extremist movements: the role of ideological contagion

Terrorism and the rise of extremist movements are further examples where PC plays a critical role in ideological transmission. Studies have shown that emotional contagion (especially fear and anger) and perceptual contagion (i.e., how certain ideologies become cognitively salient) are key drivers in the recruitment process to extremist ideologies (Horgan, 2008). Terrorist organizations, for example, often use emotionally charged narratives and propaganda that invoke strong feelings of injustice or fear, which are then propagated through social networks, fueling further radicalization.

The social contagion of extremist beliefs is often sustained by individuals’ desire for social belonging and identity reinforcement. As individuals become part of extremist groups, they increasingly adopt shared beliefs and values, mirroring the emotional and ideological stance of the group. These shared, emotionally charged narratives not only propagate extreme views but also provide a sense of purpose, belonging, and identity, which reinforces the behavioral contagion of participating in violent actions or radical activities.

### 7.10 Conclusion

In each of these modern-day controversies—whether involving health scares, conspiratorial thinking, or online trolling— PC is arguably a pivotal mechanism that drives the spread of misinformation, emotional distress, and social unrest. The interactions between emotional, perceptual, and behavioral contagion in these contexts underscore the power of social influence and the role of digital platforms in amplifying personal mentations and collective behaviors. Understanding these contagion processes, particularly within the context of misinformation and online behavior, is crucial for mitigating harmful effects, fostering digital literacy, and promoting more informed and resilient social environments.

## 8 Role of contagion in anomalous experiences (AEs)

AEs involve altered, anomalous, or non-ordinary perceptions that are typically spontaneous and challenge percipients’ assumptions about the nature of reality or their place in it (e.g., Chirico et al., 2022). PC-related effects also have been implicated in the emergence and transmission of various AEs, including trance states, mystical visions, possession episodes, and haunt-poltergeist episodes. These phenomena, often reported in religious, spiritual, or culturally specific contexts, highlight the permeability of perceptual and cognitive boundaries under the influence of social and affective cues (e.g., Houran, 2000). In such settings, contagion processes may heighten individual suggestibility, blur distinctions between self and other, and facilitate the uptake of shared cognitive-emotional frameworks that support anomalous or paranormal interpretations.

Group-based rituals, for instance, often involve rhythmic chanting, synchronized movement, or emotional arousal—conditions known to fuel both emotional and perceptual contagion (Atkinson, 1992; McNeill, 1995). These states can facilitate psychological absorption or other altered states of consciousness, allowing participants to perceive visions, healing effects, or spirit encounters that conform to shared cultural templates (Cardeña, 2011). The phenomena of speaking-in-tongues (glossolalia), mass trance, and religious ecstasy can thus be viewed as culturally-structured expressions of PC operating through emotional entrainment and suggestive framing (Goodman, 1988; Lewis, 2003).

Anthropological and historical analyses of “psychic epidemics” further demonstrate how anomalous experiences can spread in clustered, socially patterned ways. Medieval dancing plagues, spirit possession outbreaks, and Marian apparitions have been interpreted as collective expressions of distress, suggestibility, and belief amplification within tightly bonded communities (Colligan et al., 1982; Bartholomew and Wessely, 2002). In many such cases, the content of the anomalous experience reflects dominant cultural narratives—whether demonic, divine, or conspiratorial—while the contagion itself is mediated by emotional resonance, shared expectation, and rapid social communication.

Even in contemporary clinical contexts, the social framing of anomalous experience can influence its course and interpretation. Research in cultural psychiatry and transpersonal psychology suggests that individuals who report hearing voices, experiencing telepathy, or sensing non-local presences often do so in relation to emotionally charged interactions or group affiliations (Jackson and Fulford, 1997; Luhrmann, 2011). When such experiences are socially validated—as in religious or spiritual groups—they may be integrated into identity without distress; when invalidated or pathologized, they may contribute to clinical symptomatology (Peters et al., 1999).

The contagious spread of interpretive frameworks is especially salient in contexts involving paranormal media (e.g., Hill et al., 2018), conspiracy theorist communities (as discussed above), religious groups reporting collective experiences (e.g., Bennett, 2012), or “flaps” of UFO/UAP sightings (e.g., Gow et al., 2001), where anomalous perceptions may be reinforced by group feedback, digital algorithms, or memetic propagation (Childs and Murray, 2010; French and Stone, 2014; Harambam and Aupers, 2015; Hill et al., 2018; Drinkwater et al., 2019; Eaton, 2019). Here, PC enables anomalous beliefs or experiences to crystallize through repeated exposure, confirmation bias, and social reinforcement—aligning with broader models of expectancy-driven perception (Schwarz, 1994). Together, these findings point to a broader interpretation of PC—not merely as the transfer of emotions or behaviors, but as a potent mechanism by which subjective reality itself becomes socially constructed, distributed, and sustained. Understanding how AEs propagate within group settings provides a unique vantage point on the cognitive-affective mechanisms underlying both personal transformation and collective belief formation (see e.g., Eaton, 2019; Langston and Hubbard, 2019; Ironside and Wooffitt, 2021).

For example, “(entity) encounter experiences” often involve contextual variables that can prime or cue percipients (Houran, 2000) or can involve fear-induced feedback loops that sustain experiences (Lange and Houran, 1999). Several empirical studies indeed validate the power of suggestion-expectancy effects for inducing AEs (French, 1992; Smith, 1992–1993; Lange and Houran, 1997; Wiseman et al., 2003; French et al., 2009; Simmonds-Moore et al., 2017), and such effects in quasi-experimental settings have been observed to fuel snowballing perceptions within individuals or across a group of people akin to a viral outbreak (e.g., Houran and Lange, 1996; Laythe et al., 2017). Moreover, time-series analyses of the onset of discrete AEs in some spontaneous cases reveal marked “flurries or clusters” of events consistent with PC effects (Lange and Houran, 2001a,b; Houran et al., 2022).

These types of findings, for instance, have led Houran et al. (2002, 2022, 2023, 2024) to interpret haunt-poltergeist episodes (and their associated concept of Haunted People Syndrome; cf. Laythe et al., 2021, 2022) as a form of MPI (see e.g., Houran and Lange, 1996; Lange and Houran, 2001a; O’Keeffe et al., 2019, 2025; Lange et al., 2020; Houran and Laythe, 2022; Dagnall et al., 2025). That said, statistical studies have also analyzed the averaged published prevalence rates of certain AEs against the averaged published effect sizes of suggestion-expectancy and related PC effects and found a clear gap, which implies that the influence of PC mechanisms alone might not account for various types of AEs (Laythe and Houran, 2022; Rock et al., 2023).

## 9 Collective insights and future research directions

When examining the full array of PC-related phenomena, several cross-cutting insights and patterns emerge that isolated studies do not always emphasize:

### 9.1 Contagion cascades are often multi-modal

Many large-scale events involve sequential or concurrent activation of emotional, perceptual, and behavioral contagion. In mass hysteria, for example, emotional anxiety spreads (emotional contagion), leading to symptom perception (perceptual contagion), followed by mimicked or enacted behaviors (behavioral contagion) (cf. Ali-Gombe et al., 1996). This suggests that contagion types often scaffold each other, forming cascading chains across modalities. This cascading pattern typically reflects Mechanism 1: Suggestibility → Mechanism 3: Automatic mimicry → Mechanism 9: Feedback loops and threshold dynamics (see Table 3).

### 9.2 Group amplification hinges on shared identity and synchronization

Group-oriented contagion effects—especially mass conversion reactions, mob behavior, and moral outrage—are most potent when (a) There is shared identity (e.g., national, religious, ideological); (b) Attention is synchronized (e.g., through media or physical proximity); and (c) Emotional cues are highly salient or moralized. These are the conditions under which the “cascading-resonance” effect becomes most visible: individual-level cues get magnified into collective outcomes. These conditions instantiate Mechanism 4: Attention synchronization and Mechanism 5: Interpersonal entrainment, amplified by Mechanism 7: Network and institutional amplifiers (see Table 3).

### 9.3 Digital environments blur person- and group-oriented boundaries

In online contexts, individual contagion can scale instantly to group effects, and group-level contagion (e.g., a viral post) can rapidly impact individuals. Algorithms act as super-spreaders, intensifying feedback loops and creating artificial critical masses (e.g., via trending or boosting emotionally charged content). This has led to emergent phenomena like (a) “Memeplex contagion” (bundled behaviors or ideologies spreading through memes); and (b) “Algorithmic suggestion bias” (suggestion-expectancy effects driven by curated exposure). Lange and Houran (2000) found that beliefs in exceptional (paranormal) events follows a fold catastrophe model where the acceptance or non-acceptance of such beliefs can be seen as two mutually exclusive states that are fueled by respondents’ levels of involvement. Since curated exposure tends to increase readers’ involvement in the underlying events, their model explains why social media heighten the intensity and polarity of viewpoints expressed online. This dynamic is driven by Mechanism 7: Network and institutional amplifiers together with Mechanism 4: Attention synchronization and Mechanism 9: Feedback loops (see Table 3).

### 9.4 Suggestibility is a cross-domain unifier

Suggestibility—not just cognitive but affective and behavioral—emerges as a central variable across all forms: (a) It drives susceptibility to placebo/nocebo (perceptual); (b) Enhances mimicry or mirroring (emotional); and (c) Increases conformity or imitation (behavioral). Thus, suggestibility may be the central psychological substrate that links all contagion forms—regardless of content (cf. Sapkota et al., 2019, 2020). Research has consistently shown that individuals with more permeable mental boundaries—often measured through constructs such as transliminality, suggestibility, and dissociative tendencies—are more susceptible to PC effects. Transliminality, defined as a heightened sensitivity to internal and external psychological stimuli, has been associated with increased absorption, fantasy proneness, and a tendency to blur the distinction between self and environment, which can enhance receptivity to external emotional or behavioral cues (Evans et al., 2019; Merckelbach et al., 2022; Rosen et al., 2023; Roxburgh et al., 2024; Simmonds-Moore, 2024; Swami et al., 2024; cf. Palsson, 2025). This heightened openness may facilitate the uncritical internalization of others’ perceptions, emotions, interpretations, or actions, which are hallmarks of PC.

Similarly, individuals high in suggestibility are more likely to accept and internalize others’ suggestions, which increases their vulnerability to contagious emotional or behavioral states (e.g., Cardeña et al., 2008; Lynn et al., 2008). Suggestibility frequently overlaps with transliminality and has also been linked to proneness for AEs, including the subjective absorption of social and emotional content. Dissociative tendencies—such as *depersonalization* (i.e., a feeling of detachment from one’s own thoughts, feelings, body, or actions) and *derealization* (i.e., a feeling of detachment or unreality regarding the external world, where individuals perceive their surroundings as strange, dreamlike, or distorted)—further contribute to the permeability of mental boundaries by weakening the integration of thoughts, feelings, and experiences. This fragmentation can enhance responsiveness to emotionally charged social environments, heightening susceptibility to contagion processes (Evans et al., 2019).

Together, these traits form a psychological profile marked by “thin mental boundaries” (Hartmann, 1991; Evans et al., 2019; Lange et al., 2019), which ostensibly predisposes individuals to emotional, perceptual, or behavioral forms of PC. Such findings are relevant in understanding the mechanisms behind mass hysteria, social mimicry, and collective emotional shifts. Measured by Hartmann’s (1991) Boundary Questionnaire, the Revised Transliminality Scale (Lange et al., 2000), or Suggestibility Scales (Acunzo and Terhune, 2021; Stein et al., 2025a), Sensory-Processing Sensitivity, and related perceptual-personality variables like Intolerance of Ambiguity and Aberrant Salience. Perhaps the measures of emotion contagion can be augmented with items re: transliminality, etc., for a robust, inclusive assessment tool for PC. Nevertheless, suggestibility overall functions as Mechanism 1: Suggestibility and boundary thinness, moderating Mechanisms 2–6 across domains (see Table 3).

### 9.5 Contagion can be spontaneous or engineered

Some contagion effects are emergent and self-organizing (e.g., laughter in a crowd), whereas others are strategically induced for influence or manipulation. For instance, viral marketing, propaganda, “psy-ops” (i.e., psychological operations or a military strategy aimed at influencing the behavior of target audiences via information campaigns, propaganda, or deception), and even evangelism seem intentionally to exploit contagion principles. These cases often rely on emotional salience, repetition, and credibility cues, aligning closely with behavioral science and persuasion models (Cialdini, 2001). We think that Engineered spread typically leverages Mechanism 2: Expectancy and framing and Mechanism 7: Network amplifiers to create rapid cascades (Mechanism 2 → Mechanism 7).

### 9.6 Moralization is a powerful amplifier

When emotional or behavioral contagion is moralized (e.g., climate activism, cancel culture, outrage), spread becomes more contagious and more resistant to disconfirmation. This suggests that moral framing may be a key contagion intensifier, explaining why some behaviors spread virally and others do not. Moralization directly corresponds to Mechanism 8: Moralization and affective intensity and increases resistance to corrective information via Mechanism 9: Feedback loops (Mechanism 8 → Mechanism 9).

### 9.7 Contagion thresholds mirror epidemiological patterns

Many group-oriented contagion effects follow threshold or tipping point dynamics. Small influences build until a critical mass is reached, after which change accelerates nonlinearly. This aligns with network theory and diffusion of innovation models (Granovetter, 1978; Gladwell, 2000). In our view, threshold dynamics should be indexed to Mechanism 9: Feedback loops and threshold dynamics (see Table 3).

### 9.8 Contagion is context-dependent

The same mechanisms can lead to (a) Destructive outcomes (e.g., mass shootings, panic buying), and (b) Prosocial outcomes (e.g., gratitude campaigns, acts of kindness). Thus, contagion is morally neutral but ethically potent—it depends on what spreads, through whom, and under what conditions. Contextual moderators arguably operate through Mechanism 6: Social appraisal and Mechanism 1: Suggestibility to bias direction and valence of spread (Mechanism 6 ← Mechanism 1).

These latter insights suggest that PC is a complex, multi-level phenomenon that might resist explanation by any single principle or mechanism. Emotional, perceptual, and behavioral forms often operate in cascading sequences, shaped by social identity, synchronized attention, and increasingly, digital infrastructures. Shared psychological traits—such as suggestibility, transliminality, and dissociation—consistently predict susceptibility, while moralization and algorithmic amplification intensify spread and resistance to disruption. These patterns call for an integrated, cross-disciplinary approach that connects individual vulnerability, contextual triggers, and structural amplifiers. The following research directions identify key priorities for building a more comprehensive and applicable science of contagion:

#### 9.8.1 Individual differences

Future studies should prioritize studies that operationalize Mechanism 1: Suggestibility and Mechanism 5: Interpersonal entrainment (e.g., measure transliminality; record physiological synchrony). This involves the investigation of psychological and neurobiological factors that influence susceptibility to contagion. Traits such as neuroticism, empathy, and suggestibility (Doherty, 1997; Dezecache et al., 2013), alongside neurobiological markers (e.g., activity in the mirror neuron system or stress-response circuits; Wicker et al., 2003; Keysers and Gazzola, 2009), may help to identify who is most vulnerable to contagion effects.

#### 9.8.2 Digital environments

The algorithmic architecture and social dynamics of digital platforms play an increasingly significant role in the spread of emotional and behavioral contagion (Kramer et al., 2014; Brady et al., 2020). Longitudinal research and machine learning approaches are particularly well-suited to detect propagation patterns, feedback loops, and contagion tipping points in online environments (Sheetal et al., 2023). We specifically suggest developing longitudinal models that target Mechanism 7: Network and institutional amplifiers and Mechanism 4: Attention synchronization to identify “tipping points” or “critical mass” effects.

#### 9.8.3 Interventions and resilience

A critical research priority involves identifying strategies to buffer contagion (Bonanno, 2004) and mitigate its potentially harmful effects—such as anxiety, panic, and self-harm—while fostering “positive contagion” including prosocial behavior and collective wellbeing (Fredrickson, 2001; Singhal and Rogers, 2003; Miller and Kelly, 2020). This entails designing interventions that reduce Mechanism 4: Attention synchronization (decrease algorithmic salience) and dampen Mechanism 9: Feedback loops (reduce reinforcement signals). Insights from this line of inquiry could inform public health messaging, clinical interventions, and social media platform design (Southwick and Charney, 2018).

#### 9.8.4 Cross-cultural perspectives

Emotional and behavioral contagion are shaped by cultural norms governing expression, regulation, and social interpretation (Mesquita and Walker, 2003; Tsai et al., 2007). Comparative and cross-cultural studies can help to distinguish universal mechanisms from culture-specific dynamics, broadening the ecological validity of contagion models.

#### 9.8.5 Neurocognitive mechanisms

Advances in neuroimaging and psychophysiological recording provide opportunities to explore the real-time neural and bodily correlates of contagion (Decety and Lamm, 2006; Gallese, 2007). Such approaches can clarify causal mechanisms, including the roles of embodied simulation, attentional tuning, and affective resonance. For instance, hyperscanning might be effective to test Mechanism 5: Interpersonal entrainment as a causal mediator of behavioral alignment.

#### 9.8.6 Intersections and overlaps

Future work should examine the co-occurrence and interaction of different contagion types—particularly emotional and perceptual contagion—across contexts such as mass hysteria, mediated communication, and clinical presentations (Hatfield et al., 1993a,b; Bartholomew and Wessely, 2002).

#### 9.8.7 Conceptual tensions

Competing explanatory frameworks—such as cognitive versus embodied models, or culturally sensitive psychiatry versus universal neurobiological generalizations—point to unresolved theoretical tensions (Kleinman, 1988; Choudhury and Slaby, 2012). Research that explicitly tests these frameworks can help to integrate or differentiate their explanatory power.

#### 9.8.8 Underexplored issues

Several areas remain underdeveloped but are critical for a fuller account of PC: (a) The temporal dynamics and maintenance mechanisms of contagion across time (Houran and Lange, 1996; Lange and Houran, 2001a; Collins, 2004); (b) Non-human analogues such as swarm behavior or animal mimicry as models for contagion processes (Sumpter, 2011); and (c) Linguistic and narrative factors—including semantic priming and story framing—that shape susceptibility to implicit or explicit cues (Drinkwater et al., 2019; Green and Brock, 2000; Mattila, 2000).

PC operates at the intersection of individual psychology, social dynamics, cultural systems, and neurobiological processes. While significant advances have illuminated certain mechanisms and manifestations, the field remains conceptually fragmented and empirically uneven. Future research should adopt interdisciplinary frameworks, integrate culturally diverse perspectives, and prioritize real-world applicability—particularly in the domains of digital media, public health, and collective behavior. A more comprehensive science of PC-related phenomena will depend not only on identifying *what* spreads, but on understanding *who* spreads it, *why* it spreads, and *how* it might be constructively channeled or constrained.

## 10 An integrative “Cascading-Resonance Model” of PC phenomena

Grounded theory is a research method for developing a model or explanatory framework directly from data gathered in real-life observations and experiences; rather than beginning with a hypothesis, researchers iteratively collect and analyze data so that patterns and insights emerge organically, with the resulting theory said to be “grounded” in what was observed (Bryant, 2017). Such analysis nonetheless involves a degree of bias related to researcher interpretation, analytic choices, and mapping judgments—all of which can affect findings, so transparency and reflexivity aim to enhance the credibility of the resulting framework.

Based on the evidence and insights gleaned from our thematic mapping and review, we propose a three-layer, cascading-resonance model in which discrete, numbered mechanisms operate sequentially and interactively to produce contagion at scale (see Table 4 for layer–mechanism–indicator mapping). At the *micro-level*, PC commonly originates in individual resonance, where an observer internally mirrors another’s emotion, perception, or action via affective empathy, automatic mimicry, or narrative transportation; this input layer is instantiated primarily by Mechanism 1: Suggestibility and Mechanism 2: Expectancy and framing and is frequently seeded by Mechanism 3: Automatic mimicry (Decety and Jackson, 2004; Cardeña et al., 2008). Individual resonance lowers thresholds for social influence, making priors, cues, and dispositional boundary thinness central determinants of whether social signals become personally experienced states.

**TABLE 4:** Mechanism mapping for the Cascading-Resonance Model of psychological contagion.

Level	Core mechanisms	Description	Illustration	References
Individual resonance	Mechanism 1: Suggestibility; Mechanism 2: Expectancy and framing; Mechanism 3: Automatic mimicry	Micro-level internal mirroring of another’s emotional, perceptual, or behavioral state via empathy, mimicry, or suggestion.	A viewer becomes anxious after repeatedly seeing fearful expressions in a news report or social media feed.	Hatfield et al., 1993a
Interpersonal synchronization	Mechanism 4: Attention synchronization; Mechanism 5: Interpersonal entrainment; Mechanism 6: Social appraisal	Meso-level mutual reinforcement among individuals through shared attention, emotional alignment, or co-perception, often amplified in real-time interactions.	Audience members crying in unison during a live concert, or users sharing similar emotional responses to a viral video in the comments section.	Dumas et al., 2010
Group cascade	Mechanism 7: Network and institutional amplifiers; Mechanism 8: Moralization and affective intensity; Mechanism 9: Feedback loops and threshold dynamics	Macro-level propagation of psychological states across large populations, often facilitated by shared identity, cultural scripts, and digital amplification.	Outrage spreading rapidly across a nation following a politically charged event, mobilized by hashtags and influencer commentary.	Christakis and Fowler, 2013

When multiple individuals in a shared temporal, spatial, or digital context undergo similar resonance, their responses begin to cohere into interpersonal synchronization through shared attention and temporal coupling. This *meso-level* alignment is driven chiefly by Mechanism 4: Attention synchronization and Mechanism 5: Interpersonal entrainment and is conferred social meaning by Mechanism 6: Social appraisal (Dumas et al., 2010; Parkinson, 2011; Hasson et al., 2012). Empirical work indicates that attention gating increases the probability that automatic mimicry will recruit physiological and neural coupling across interactants, and that appraisal processes translate aligned affect or perception into coordinated normative action.

If interpersonal alignment is sustained, structural forces can stabilize and amplify local synchrony into population-level cascades. At this *macro-level*, Mechanism 7: Network and institutional amplifiers extend reach, Mechanism 8: Moralization and affective intensity heighten transmissibility and resistance to correction, and Mechanism 9: Feedback loops and threshold dynamics produce nonlinear tipping into sustained collective states (Christakis and Fowler, 2013; Brady et al., 2020). In digitally mediated environments, algorithmic recommendation, prestige signaling, and institutional scripting commonly instantiate Mechanism 7, while moral framing and high arousal instantiate Mechanism 8; together these mechanisms accelerate reinforcing feedback that pushes systems past contagion thresholds. The domain summaries (Emotional, Perceptual, Behavioral subsections) provide the empirical instances summarized above and map directly onto the input, synchronization, and scaling layers, respectively (see domain summaries and Table 4).

Table 4 summarizes core mechanisms, representative empirical indicators, and plausible intervention levers mapped to each model layer. These three layers form an integrated process: for example, an index frame that biases expectations (Mechanism 2) can activate susceptible observers (Mechanism 1), producing rapid mimicry (Mechanism 3) that concentrates group attention (Mechanism 4), yields interpersonal entrainment (Mechanism 5), is interpreted via social appraisal (Mechanism 6), is amplified by institutional or algorithmic forces (Mechanism 7), becomes moralized to increase arousal (Mechanism 8), and is then driven across a tipping point by reinforcing feedback (Mechanism 9). This chain illustrates how individual-level receptivity and micro-interactional dynamics combine with meso- and macro- level amplifiers to produce cascading resonance across emotional, perceptual, and behavioral domains. A complementary formal model shows that signed (trust/distrust) ties and the balance of pairwise versus group coupling determine whether emotional contagion produces abrupt, bistable, hysteretic shifts or instead spreads smoothly across a network (Ma et al., 2025).

The mechanism-level mapping yields testable propositions and intervention priorities. Interrupting attention synchronization (Mechanism 4) should reduce transitions from individual resonance to interpersonal alignment; constraining network amplification (Mechanism 7) should limit large-scale cascades even when input- and synchronization-level mechanisms are present; and reducing moralization or affective intensity (Mechanism 8) should shorten cascade persistence and increase receptivity to corrective information. These propositions translate directly into measurable manipulations and potential policy levers.

Measurement guidance follows from the proposed layered architecture: input-layer measures might include transliminality, ECS/CAPS scores, priming effects, and facial EMG to test Mechanisms 1–3; synchronization-layer metrics might include hyperscanning synchrony, shared gaze, and autonomic covariation to test Mechanisms 4–6; and scaling-layer indicators might include algorithmic trending metrics, adoption curves, and the prevalence of moralized discourse to test Mechanisms 7–9. Corresponding intervention levers include framing and inoculation at the input layer, attention diversification and normative reframing at the synchronization layer, and algorithmic dampening or delay buffers at the scaling layer. For clarity and operationalization, Table 4 also shows our mapping of the three model layers to their core mechanisms, representative empirical indicators, and plausible intervention levers.

## 11 Discussion

Our narrative review offers a broad conceptual synthesis of PC but is limited in several key respects. It relies on theoretical integration versus empirical data, making our conclusions more suggestive than conclusive. Additionally, our emphasis on illustrative examples across diverse domains—such as media, crowd behavior, and neurobiology—risks overgeneralization and may obscure important contextual differences. Our scope also precludes in-depth treatment of cultural, longitudinal, and individual difference factors that modulate susceptibility to contagion-related phenomena. Moreover, grounded synthesis depends on extant literature and coding decisions; the recent expansion of digital studies may bias perceived prominence of scaling mechanisms. The numbered mechanisms are parsimonious abstractions and may obscure culturally specific sub-mechanisms or contextual modifiers. Large scale causal identification of algorithmic amplification and threshold dynamics remains difficult and will require coordinated collaborations with platforms and regulatory partners. Finally, our first-iteration Cascading-Resonance Model lacks a detailed formal operationalization or empirical validation. The idea that discrete mechanisms work synergistically to produce larger scale PC effects therefore could be overstated.

Notwithstanding these caveats, many important insights emerged from this research. Contrary to the traditional view of consciousness as a fundamentally private phenomenon, our findings highlight the profound embeddedness of “subjective experience” in sociocultural dynamics—it might even be said that the human mind is inherently “meme-spirited” (cf. Hill et al., 2018, p. 117). The PC concept therefore presents not as a monolithic or peripheral phenomenon, but as a central mechanism through which human experience becomes shared, transmitted, and ultimately transformed. Drawing from diverse literatures in psychology, neuroscience, media studies, and cultural theory, we show that contagion operates along distinct but interacting dimensions—emotional, perceptual, and behavioral—each governed by several overlapping mechanisms.

Specifically, grounded synthesis of the selected literature set generated a coherent, mechanism-level account that organizes PC into a three-layer cascading-resonance architecture (cf. Table 4). The model distinguishes input-level processes of individual resonance (Mechanisms 1–3), meso-level processes of interpersonal synchronization (Mechanisms 4–6), and macro-level scaling processes (Mechanisms 7–9). Treating contagion as an integrated, recursive system clarifies how affective, perceptual, and behavioral phenomena can reflect the same underlying process architecture rather than disconnected metaphors.

This review establishes that individual susceptibility consistently matters. Dispositional receptivity, expectancy effects, and automatic mimicry lower thresholds for social influence and reliably predict short-term affective and perceptual convergence; experimental work that measures mimicry, priming, and susceptibility provides the strongest causal evidence for these input mechanisms. Studies of behavioral and neurophysiological synchrony substantiate the synchronization layer by demonstrating that shared attention and interpersonal entrainment convert isolated resonance into coordinated states; these findings support Mechanisms 4–6 as pathways through which individual-level effects propagate among interactants.

Evidence for scaling processes is more heterogeneous but increasingly compelling in digitally mediated contexts. Trace data and natural experiments implicate platform algorithms, prestige cues, institutional messaging, and moral framing in amplifying and stabilizing local synchrony into population-level cascades, consistent with Mechanisms 7–9. However, causal identification of these macro mechanisms remains methodologically challenging; stronger inference requires field experiments, preregistered platform interventions, and cross-platform replications.

Specifying numbered mechanisms yields three theoretical payoffs. First, it reduces conceptual ambiguity by mapping domain-specific phenomena (for example, emotional mimicry, perceptual convergence, and mass imitation) onto a single process architecture. Second, it clarifies bidirectional causality: macro signals can back-propagate to alter individual priors and receptivity, and micro processes can aggregate into macro-outcomes through recursive feedback. Third, it provides a transparent taxonomy that supports falsifiable hypotheses and cumulative comparison across studies.

The layer–mechanism mapping has direct policy and intervention implications. Interventions that target leverage points—such as reducing synchronous exposure to high-arousal content (Mechanism 4), inserting delay buffers to disrupt reinforcing feedback (Mechanism 9), or dampening moralized framing that increases transmissibility (Mechanism 8)—are likely to outperform content-only strategies. Table 4 operationalizes these inferences by linking each layer to representative indicators and practicable levers, and it should guide evaluation designs that measure proximal mechanism change rather than only distal outcomes.

We recommend a targeted research program to advance theory and practice. First, researchers should pre-register studies that operationalize and measure specific mechanisms (for example, validated scales for suggestibility and transliminality for Mechanism 1, hyperscanning paradigms for Mechanism 5, and trending/adoption metrics for Mechanism 7). Second, causal tests that manipulate transitions between layers—such as experimental modulation of attention salience to test Mechanism 4’s causal role in moving from resonance to entrainment—are particularly valuable. Third, multimethod, multilevel designs that combine self-report, behavioral observation, physiological recording, neural hyperscanning, and digital trace data will permit stronger inferences about how micro processes produce macro patterns. Finally, field experiments on platforms that implement delays, dampening, or reframing provide the most direct test of scaling mechanisms and immediate policy relevance.

Ultimately, our Cascading-Resonance Model reconceptualizes PC as a testable, mechanism-driven process that unifies affective, perceptual, and behavioral phenomena across micro, meso, and macro levels. Although provisional and awaiting empirical validation, the framework translates abstract theory into concrete, measurable indicators and actionable levers that researchers and policymakers can target. By prioritizing mechanism-targeted measurement, pre-registered causal tests, and field experiments that intervene specifically at synchronization and scaling points—such as attention diversification, algorithmic dampening, and delay buffers—we can more quickly blunt harmful cascades, amplify beneficial spread, and build a stronger causal foundation for understanding how emotional, perceptual, and behavioral influences are propagated in an era of unprecedented social interconnection and a correspondingly “entangled” constellation of minds.
